# Enhanced Antimalarial and Antisequestration Activity of Methoxybenzenesulfonate-Modified Biopolymers and Nanoparticles for Tackling Severe Malaria

**DOI:** 10.1021/acsinfecdis.3c00564

**Published:** 2024-01-25

**Authors:** Adrian Najer, Junyoung Kim, Catherine Saunders, Junyi Che, Jake Baum, Molly M. Stevens

**Affiliations:** † Department of Materials, Department of Bioengineering, and Institute of Biomedical Engineering, Imperial College London, London SW7 2AZ, U.K.; ‡ Department of Life Sciences, Imperial College London, London SW7 2AZ, U.K.; § Department of Physiology, Anatomy and Genetics, Department of Engineering Science, and Kavli Institute for Nanoscience Discovery, University of Oxford, Oxford OX1 3QU, U.K.

**Keywords:** Plasmodium, severe malaria, sequestration, biomaterial, nanoparticle, inhibition

## Abstract

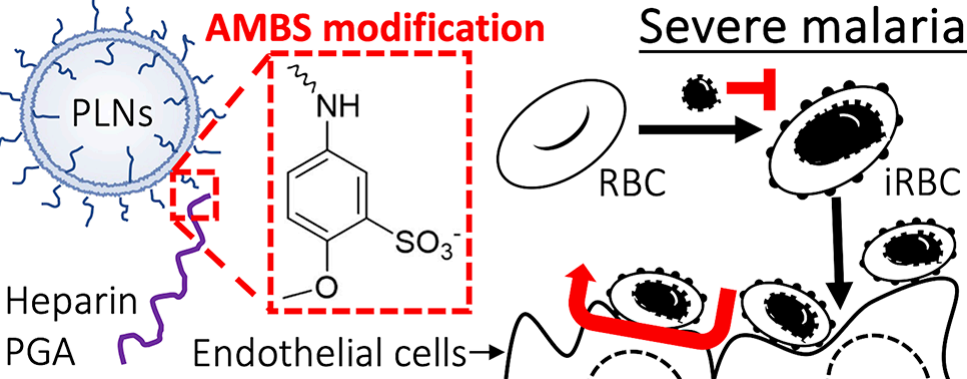

Severe malaria is a life-threatening condition that is associated with a high mortality. Severe *Plasmodium falciparum* infections are mediated primarily by high parasitemia and binding of infected red blood cells (iRBCs) to the blood vessel endothelial layer, a process known as sequestration. Here, we show that including the 5-amino-2-methoxybenzenesulfonate (AMBS) chemical modification in soluble biopolymers (polyglutamic acid and heparin) and poly(acrylic acid)-exposing nanoparticles serves as a universal tool to introduce a potent parasite invasion inhibitory function in these materials. Importantly, the modification did not add or eliminated (for heparin) undesired anticoagulation activity. The materials protected RBCs from invasion by various parasite strains, employing both major entry pathways. Two further *P. falciparum* strains, which either expose ligands for chondroitin sulfate A (CSA) or intercellular adhesion molecule 1 (ICAM-1) on iRBCs, were tested in antisequestration assays due to their relevance in placental and cerebral malaria, respectively. Antisequestration activity was found to be more efficacious with nanoparticles *vs* gold-standard soluble biopolymers (CSA and heparin) against both strains, when tested on receptor-coated dishes. The nanoparticles also efficiently inhibited and reversed the sequestration of iRBCs on endothelial cells. First, the materials described herein have the potential to reduce the parasite burden by acting at the key multiplication stage of reinvasion. Second, the antisequestration ability could help remove iRBCs from the blood vessel endothelium, which could otherwise cause vessel obstruction, which in turn can lead to multiple organ failure in severe malaria infections. This approach represents a further step toward creation of adjunctive therapies for this devastating condition to reduce morbidity and mortality.

Malaria is a mosquito-transmitted parasitic infection that caused 249 million infections and 608,000 deaths in 2022.^1^ Severe forms of the disease, responsible for these deaths, are associated with hyperparasitemia in the blood (∼2.5–20% depending on the epidemiological context) and “sticky” late-stage infected red blood cells (iRBCs) obscuring microvasculature blood flow, eventually causing serious multiple organ failure. Even with access to state-of-the-art hospitals, the mortality associated with severe malaria is still about 10–20%, and survivors of cerebral malaria can be neurologically disabled for life.^2−4^ The multiplication rate per life cycle (ca. 2 days) was found to be around 16-fold for *Plasmodium falciparum* in humans,^5^ representing a key intervention point. Another hallmark of severe *P. falciparum* infection in humans is the phenomenon called sequestration, *i.e.*, binding of iRBCs to specific receptors on the blood vessel endothelium.^6^ Hence, interfering with both invasion and sequestration might be a suitable strategy to avoid potentially fatal multiplication and blood flow obstruction in severe malaria cases.

Parasites achieve sequestration through mutually exclusive expression of 1 out of 60 *var* genes encoding *P. falciparum* erythrocyte membrane protein 1 (*Pf*EMP1). This protein is translocated to the iRBC surface to mediate specific interactions with endothelial receptors including heparan sulfate,^7,8^ chondroitin sulfate A (CSA),^9^ and intercellular adhesion molecule 1 (ICAM-1),^10^ among others.^6^ The CSA interaction can cause placental malaria, while the ICAM-1 interaction is associated with cerebral malaria; both represent severe health risks for the unborn child and mother and cerebral malaria patient, respectively. Adjunctive therapeutic measures that can potently reverse these interactions have been sought for a long time and are urgently required to reduce mortality. This is particularly the case because iRBCs continue to sequester in the blood microvessels, even after killing the intracellular parasites with standard antimalarials.^11^

Heparin has a long-standing history in evaluation for severe malaria treatment because heparan sulfate on endothelial cells serves as a receptor for *Pf*EMP1, which is part of the knoblike protrusions on the iRBC surface. Binding of heparin with *Pf*EMP1 is mediated through lysine-rich, cationic regions of *Pf*EMP1, called the Duffy-binding-like domain 1 (DBL-1),^7,8^ and single-molecule force spectroscopy determined a binding force of about 28–46 pN.^12^ However, heparin use was banned for severe malaria application after initial trials in the 1970s due to serious side effects, such as internal bleedings caused by its inherent anticoagulation activity.^13,14^ This has led to a breadth of research aimed at reducing the anticoagulation properties of heparin or heparin-like molecules, while keeping the invasion inhibitory and antisequestration functions to treat infections.^15−18^ One formulation (sevuparin) has progressed up to *in vivo* trials in humans but revealed only a transient desequestration effect and some activity against merozoite invasion, clearly requiring further optimization to be a valuable adjunctive treatment option for severe malaria.^16^ Unfortunately, heparin-based structures, even when modified, still suffer from low specificity, low potency, and often short circulation times.^15^

Other strategies with higher target specificity that are being evaluated preclinically against sequestration in severe malaria include antibodies,^19,20^ aptamers,^21^ and small molecule inhibitors.^22,23^ However, specificity in the context of antisequestration application could mean that it might be necessary to develop and test several different inhibitors due to the 60 *Pf*EMP1 variants. It might also require detecting the dominant *Pf*EMP1 expressed in iRBCs of a patient, and then apply an inhibitor specific to that *Pf*EMP1 variant, which could cause the parasites to switch to another *Pf*EMP1, subsequently avoiding the inhibitor. Hence, finding more broadly applicable therapies that act against various *Pf*EMP1 variants, while providing both sequestration inhibition and reversal action, would be of benefit.^24^

Nanotechnology has recently become more prominent in various applications against infectious diseases, including for diagnostic, therapeutic, and preventive interventions.^25^ Indeed, nanoparticles are also an upcoming tool for improving efficacy of existing antimalarials through targeted delivery^26,27^ and as invasion inhibitors.^15,28−30^ In the search for a simple nanoparticle system for the latter application, we have recently shown potent *Plasmodium* invasion inhibition with synthetic polymer–lipid hybrid nanoparticles (EC_50_ at 0.38 ± 0.16 nM particle concentration) that functioned by direct merozoite binding after egress *in vitro* and in a mouse malaria model.^30^ We further demonstrated circulation time increase by incorporating poly(ethylene glycol)-modified (PEGylated) lipids, but antisequestration activity has not yet been evaluated for this system. In the specific case of severe malaria mitigation, only a few nanoparticle-based strategies have yet been studied and with a focus on drug delivery rather than antisequestration.^2,31^ Recently, nanoparticles were coated with brain microvascular endothelial cell (BMEC) membranes to allow binding to iRBCs and target brain microvasculature, combined with controlled release of drugs for treating cerebral malaria.^32^ However, no direct evidence of antisequestration activity against *P. falciparum* was demonstrated as of yet for these nanoparticles. Further, finding more easily translatable solutions, without the use of complex biological components such as native cell membranes, might be beneficial.

Here, we chemically modified soluble biopolymers (polyglutamic acid (PGA) and heparin) with the same chemical unit (5-amino-2-methoxybenzenesulfonic acid, AMBS) used in our previous nanoparticle study.^30^ This modification transformed PGA into an antimalarial polymer with a high potency. Furthermore, modifying the free carboxylic acids within heparin also increased antimalarial activity, while the undesired anticoagulation activity was nearly completely abolished. Both biopolymers showed no cytotoxicity when tested at relevant concentrations with a macrophage-like cell line. Besides evaluating parasite growth inhibition potential of these soluble biopolymers and our previously developed nanoparticles,^30^ we sought to test antisequestration ability, which would be of great benefit as an adjunctive therapy for severe malaria treatment. Both PGA-AMBS and nanoparticles were found to be potent inhibitors of iRBC sequestration, with the nanoparticles exhibiting even higher activity than gold-standard soluble CSA. We demonstrated this activity with the parasite lines CS2, modeling the severe pathology of placental malaria, and ItG, which binds ICAM-1 and is associated with deadly cerebral malaria. We finally confirmed the activity of our nanoparticles in prevention and reversal of ItG iRBCs binding to ICAM-1 overexpressing human umbilical vein endothelial cells (HUVECs), after tumor necrosis factor α (TNF-α) stimulation. Overall, our modified biopolymers and nanoparticles serve as a basis for the development of a severe malaria adjunctive treatment, which would be highly desirable to bring down the high mortality associated with this critical medical condition.

## Results and Discussion

### Synthesis and Characterization of Methoxybenzenesulfonated Materials

**Scheme 1:**
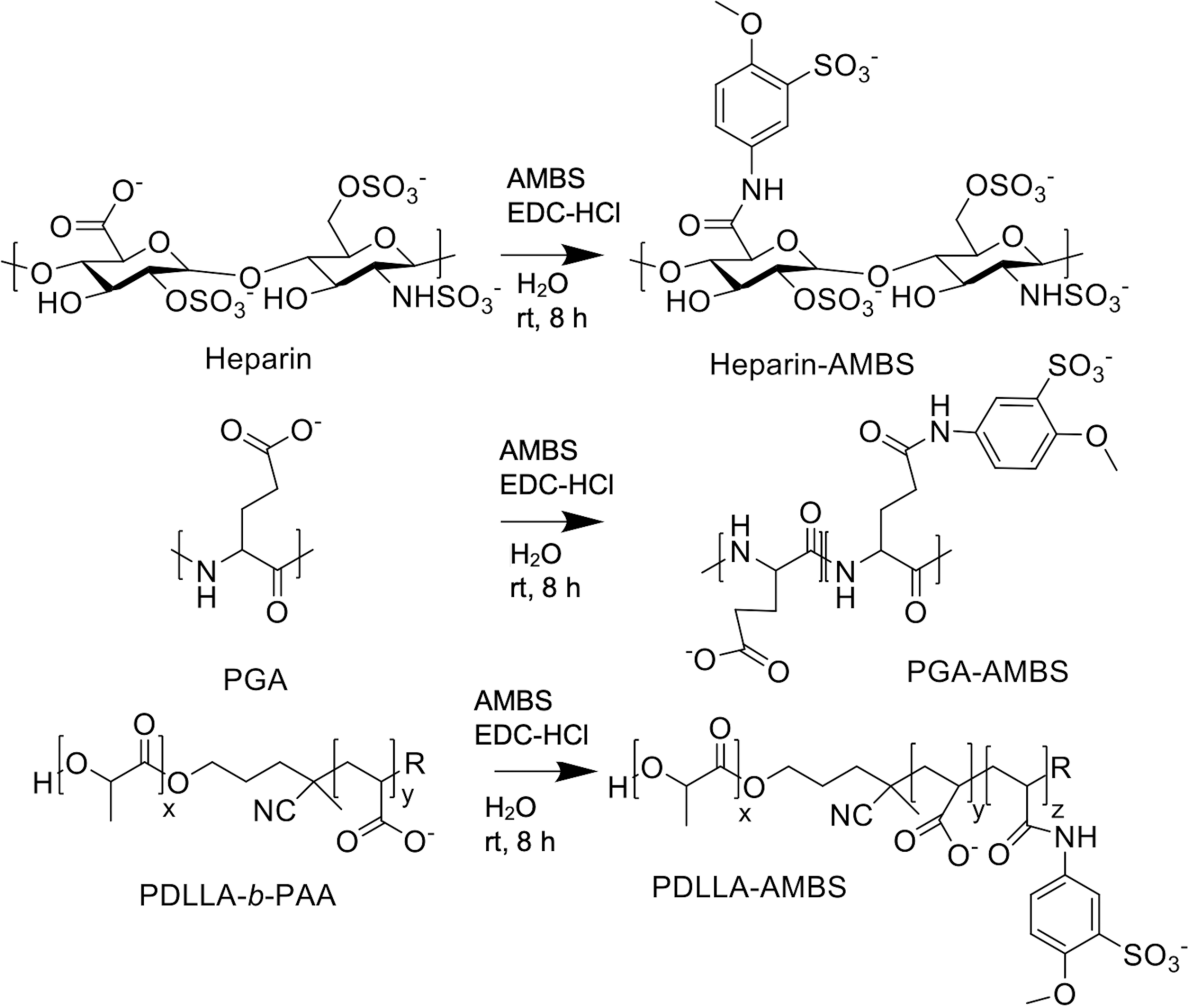
Chemical Modification of Soluble Biopolymers and Nanoparticles with AMBS Heparin, polyglutamic acid (PGA), and amphiphilic copolymer poly(d,l-lactide)-*block*-poly(acrylic acid) (PDLLA-*b*-PAA, assembled as micelles)^30^ were modified with 5-amino-2-methoxybenzenesulfonate (AMBS) in aqueous conditions (buffered at pH 6.0) using *N*-(3-dimethylaminopropyl)-*N*-ethylcarbodiimide hydrochloride (EDC-HCl)-mediated coupling at room temperature (rt) for 8 h and subsequent purification by aqueous size-exclusion chromatography (SEC). For heparin, only the major disaccharide unit is shown.

Various soluble polymers, including different versions of heparin and many other polysaccharides, have a long history of evaluation as antimalarial agents.^15−18^ However, their typically low potency and unwanted side effects, such as anticoagulation activity, have hampered their further development. Here, we evaluated modification of soluble biopolymers, including a polypeptide and heparin, with a sulfonated molecule 5-amino-2-methoxybenzenesulfonic acid (AMBS) that we have previously identified to produce potent nanoparticle-based inhibitors.^30^ Free carboxylic acids within PGA and heparin were modified similarly to the previous nanoparticle modification through *N*-(3-(dimethylamino)propyl)-*N*-ethylcarbodiimide hydrochloride (EDC-HCl)-mediated carboxylic acid activation and *in situ* amine coupling (Scheme 1). These two modified biopolymers (PGA-AMBS and Hep-AMBS) were used as soluble biopolymers and not in a nanoparticle formulation.

**Figure 1:**
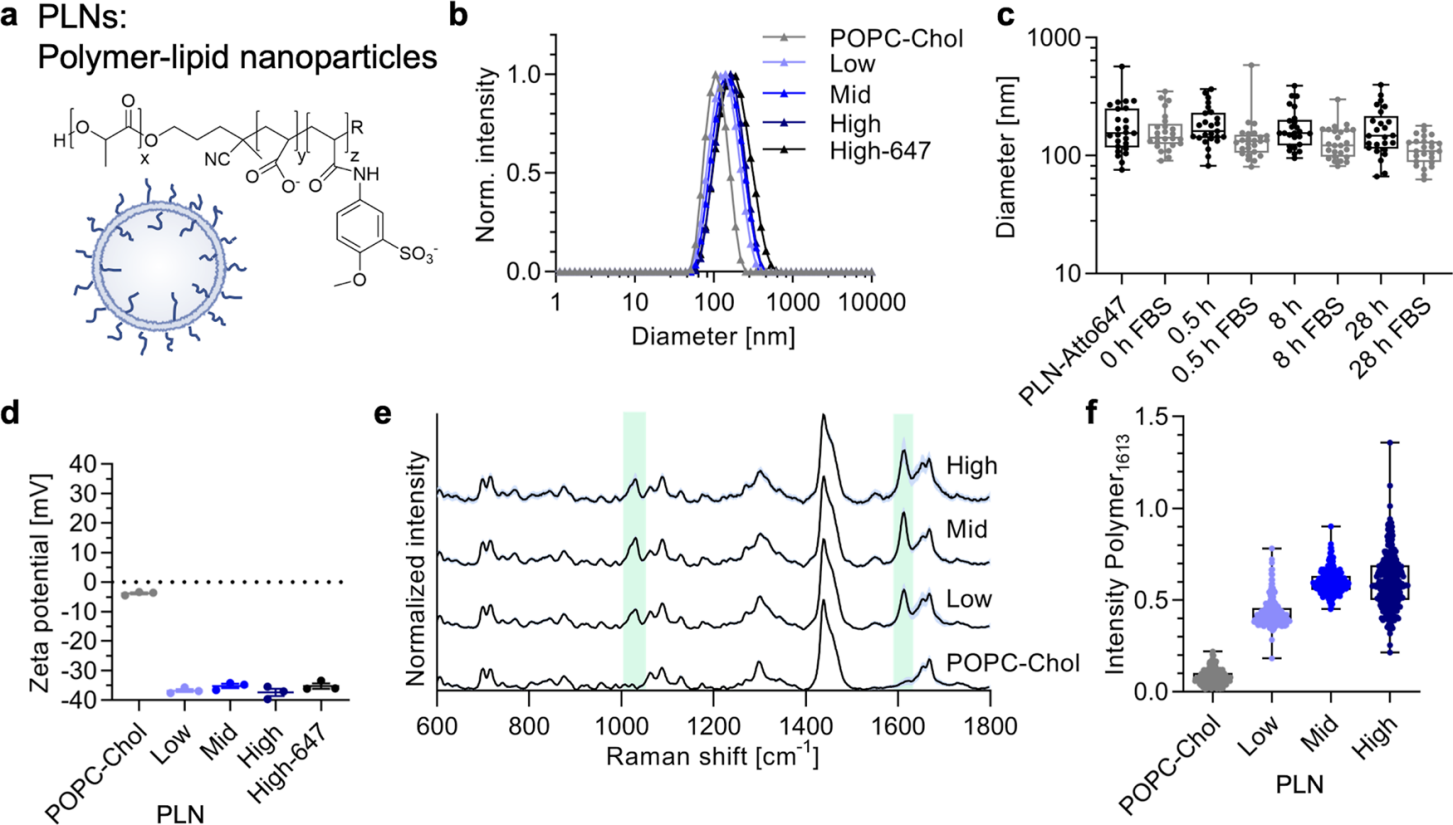
Polymer–lipid nanoparticles (PLNs) characterization. (a) Schematic of nanoparticles that consist of a mixture of lipids (POPC and cholesterol) together with the copolymer PDLLA-AMBS (chemical structure depicted). (b) Average DLS size distribution (intensity) of POPC-Chol control vesicles and nanoparticles with increasing amounts of PDLLA-AMBS (mean of technical triplicates). (c) Stability of fluorescent PLN-Atto647 over time in ±10% (v/v) FBS determined using FCS (*n* = 25 technical replicates). (d) Average ζ-potential values for samples in panel (b) (mean ± s.d., technical triplicates). (e) Normalized Raman intensities of single-particle traps as obtained by SPARTA (*n* ≥ 244, mean (black) ± s.d. (light blue)). The copolymer PDLLA-AMBS signals correspond to the sulfonate and amide/aromatic overlapping regions, respectively (1032 and 1613 cm^–1^), which are shaded in light green. (f) Raman intensity of the polymer-1613 cm^–1^ region relative to the 1439 cm^–1^ CH_2_/CH_3_ peak (from e, each dot represents one trapped particle).

The purified biopolymers revealed the characteristic ultraviolet–visible (UV–vis) absorbance peak of AMBS that could be used to determine the degree of modification, which yielded 53 and 58% for glutamic acid units in PGA and of disaccharide units in heparin (on average about 1 COOH per disaccharide), respectively (Figure S1). ^1^H NMR revealed higher degrees of modification of 92 and 91% for PGA-AMBS and heparin-AMBS (assuming 1 available COOH per disaccharide), respectively, as expected, as for UV–vis characterization, no loss during purification was assumed (Figures S2 and S3). The hydrodynamic sizes revealed that the biopolymers remained as soluble polymers after the modification rather than assembling into nanoparticles (Hep-AMBS 4.8 ± 0.4 nm and PGA-AMBS 6.7 ± 1.6 nm; dynamic light scattering (DLS), number distribution, mean ± s.d., technical triplicates). Instead of modifying soluble biopolymers, we have previously shown that the AMBS modification can be performed on the self-assembled block copolymer poly(d,l-lactide)-*block*-poly(acrylic acid) (PDLLA-*b*-PAA, 9 kDa-*b*-9 kDa), hereafter called PDLLA-AMBS.^30^ When coassembling this modified copolymer (PDLLA-AMBS) with lipids, polymer–lipid nanoparticles (PLNs) are formed.^30^ The reader is referred to our previous paper containing additional extensive analysis with respect to particle characterization (transmission electron microscopy (TEM), cryo-TEM, small-angle neutron scattering (SANS), and fluorescence correlation spectroscopy (FCS)) and biological evaluation of this system *in vitro* and *in vivo*.^30^ Here, we provide additional PLN characterization data that are relevant to the systems used herein to treat severe malaria (Figure 1).

We prepared PLNs by coinjection of an ethanolic solution of 1-palmitoyl-2-oleoyl-glycero-3-phosphocholine (POPC), cholesterol, and PDLLA-AMBS, followed by size-exclusion chromatography (SEC) purification. We have now sought to determine the saturation point for the copolymer integrated in our PLNs by preparing particles with 4, 8, and 15 mol % of the copolymer with respect to the vesicle-forming POPC component, denoted as low, mid, and high, respectively. DLS measurements revealed monodisperse hydrodynamic diameters of about 150 nm for all of the samples after purification. Stability in PBS ± 10% (v/v) fetal bovine serum (FBS) measured by DLS (Figure S1) and FCS (Figure 1c) using PLN-Atto647 revealed no aggregation of PLNs and hence good stability in physiologically relevant conditions. ζ-potential measurements suggested that the copolymer was coassembling with the lipids, as the lipid-only control exhibited neutral charge, while the PLNs revealed negative ζ-potentials, irrespective of the amount of copolymer used (Figures 1d and S1).

We next employed single-particle automated Raman trapping analysis (SPARTA),^33^ previously used to establish single-particle variation in synthetic nanoparticles,^30,34^ to evaluate changes and distributions of polymer amount per particle incorporated in PLNs. The average Raman spectra across all of the single-particle traps (Figure 1e) show the characteristic PDLLA-AMBS signals (1032 and 1613 cm^–1^) together with the lipid signal (1439 cm^–1^), confirming coassembly of the polymer and lipid on a single-particle level. Further, studying the polymer peak at 1613 cm^–1^ shows saturation of copolymer-loading at the medium feed ratio (Figure 1f, where each dot represents one trapped and measured particle). The addition of even more copolymer (high feed ratio) did not increase the average amount of the copolymer present in PLNs and instead caused a larger variation in loading per particle. We decided to employ medium and high loading for subsequent experiments to use PLNs with high copolymer presentation, maximizing the numbers of binding sites, hence, allowing for multivalent interactions between PLNs and cells.

### Biological Evaluation of Methoxybenzenesulfonated Materials

**Figure 2:**
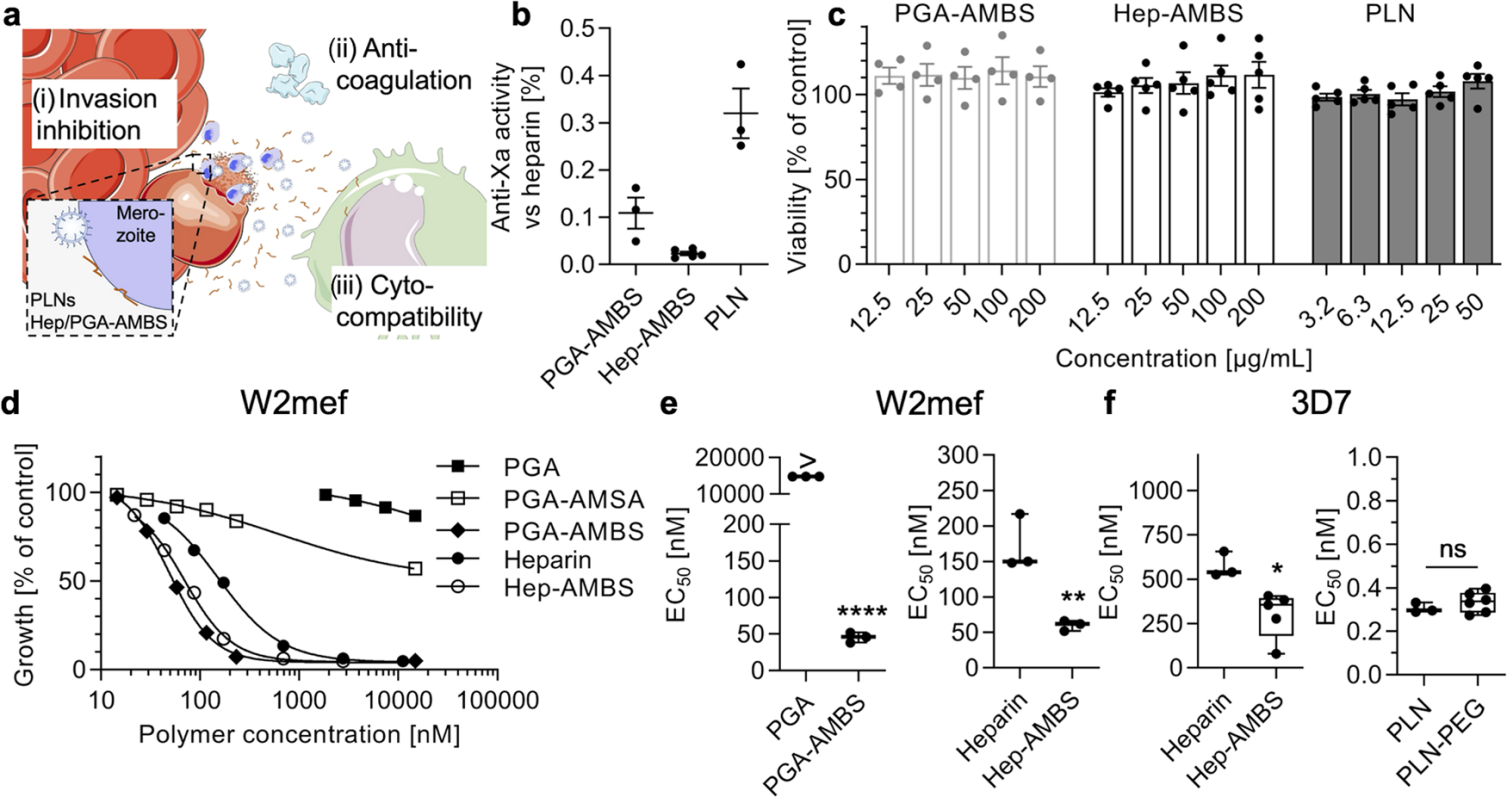
Biological evaluation of methoxybenzenesulfonated soluble biopolymers and nanoparticles. (a) Schematic summarizing the employed analysis methods. Schematic modified from Servier Medical Art Web site CC-BY. (b) Interpolated antifactor Xa relative activity when compared to unmodified heparin (100%) on a weight basis (mean ± s.e.m., *N* ≥ 3 independent experiments in duplicates). (c) Cytocompatibility of PGA-AMBS (200 μg/mL equals 9.8 μM), Hep-AMBS (200 μg/mL equals 11.1 μM), and PLNs (50 μg/mL active polymer equals 21 nM particles) when incubated with the RAW 264.7 cell line compared to PBS controls (mean ± s.e.m., *N* ≥ 3 independent experiments with technical triplicates). (d) Example dose–response curves for *P. falciparum* W2mef inhibition in suspension culture (mean and range of *N* = 1 independent experiment with technical duplicates). (e) EC_50_ values obtained from dose–response curves (as shown in panel (d)) using two *P. falciparum* strains (W2mef and 3D7) (*N* ≥ 3 independent experiments with technical duplicates, unpaired *t* tests, ns = not significant, *P < 0.05, **P < 0.01, ****P < 0.0001). Additional data with D10 and *Plasmodium knowlesi* A1-H.1 and an example dose–response curve for PLNs can be found in Figure S4. Box plots: center line, the median; box limits, upper and lower quartiles; whiskers, minimum and maximum values.

After synthesis and characterization of the AMBS-modified biopolymers and nanoparticles, we then tested their biological activity including anticoagulation activity, cytocompatibility, and invasion inhibitory potential. The anticoagulation activity of many sulfated and/or sulfonated biomaterials is a concern if the intended application is different from anticoagulation therapy. However, anticoagulation potency is often much lower than that for heparin. Polystyrenesulfonate (PSS) that is chemically related to our AMBS-modified biomaterials did not show anticoagulation activity previously;^35^ hence, we only expected little or no activity for our materials. Indeed, the AMBS modification only conferred negligible anticoagulation property to PGA and PLNs, as measured by anti-Xa tests. Only <0.5% of anti-Xa activity was observed for all of the AMBS-modified materials when compared to unmodified heparin by weight (Figures 2b and S4). This is in agreement with our previous study that found low anticoagulation property for polymer micelles based on the aminomethanesulfonic acid (AMSA)-modified copolymer (MAMSA) and AMBS-modified copolymer (MAMBS).^30^ In the case of heparin, the addition of AMBS to the carboxylic acids of the major disaccharide unit nearly completely abolished the well-known anticoagulation activity of heparin (Figure 2b). This was not unexpected since the carboxylic acid that is present in the native pentasaccharide sequence is known to be responsible for the anticoagulation activity of heparin; however, this functional group was transformed with AMBS by the modification. Modifying the carboxylic acid functional groups within heparin was previously shown to remove heparin’s interaction potential with antithrombin III, which is required for the characteristic anticoagulation property.^36^

The cytocompatibility of new therapeutics is another important characteristic. We confirmed that our modified biomaterials were cytocompatible when tested with the RAW 264.7 macrophage-like cell line at relevant concentrations (Figure 2c). In conclusion, all of our AMBS-modified biomaterials did not show significant anticoagulation potential nor were they cytotoxic. From a safety perspective, these findings confirm the suitability of our biomaterials for applications aside from anticoagulation therapy, such as the herein described antimalarial applications. We had also confirmed previously that PLNs (with 1,2-distearoyl-*sn*-glycero-3-phosphoethanolamine-*N*-[methoxy(poly(ethylene glycol))-5000], DSPE-PEG) were biocompatible *in vivo* by histological analysis after intravenous PLN injection in mice.^30^

Next, our biomaterials were evaluated in terms of *P. falciparum* invasion inhibition using various strains of the parasite known to utilize both main invasion pathways (W2mef, sialic acid-dependent pathway; 3D7 and D10, sialic acid-independent pathway). These assays involved incubating late-stage iRBCs with fresh human RBCs either with or without the inhibitor overnight and then counting the number of new-formed ring-stage iRBCs the following day using flow cytometry. Dose–response curves revealed the transformation of PGA into an antimalarial polymer by introducing the AMBS modification (PGA-AMBS, Figure 2d,e), while the unmodified PGA did not exert an inhibitory potential. Using another small amino-sulfonate molecule (AMSA) for PGA modification (PGA-AMSA) created much less potent inhibitors than the AMBS modification. PGA and PGA-AMSA were not evaluated further because they did not reveal any invasion inhibitory activity (Figure 2d). Interestingly, PGA-AMBS was even more potent than the gold-standard heparin (Table 1). This can be explained by the slightly longer polymer chain of PGA *vs* heparin combined with our previous data that larger structures tended to become better inhibitors.^28,30,37^

**Table 1:** EC_50_ Comparison from Growth Inhibition Shaking Assays against Various *P. falciparum* Strains with Sialic Acid-Dependent (W2mef) and -Independent (D10 and 3D7) Invasion Pathways (Mean ± s.e.m. of *N* ≥ 3 Independent Experiments with Technical Duplicates, from Figures 2d–f and S4)

inhibitor name	EC_50_ W2mef [nM]	EC_50_ D10 [nM]	EC_50_ 3D7 [nM]
PGA	>15,000	n/d^c^	>15,000
PGA-AMBS	45 ± 4	n/d	98 ± 38
heparin	172 ± 23	842 ± 105	573 ± 41
heparin-AMBS	60 ± 4	134 ± 10	300 ± 76
MAMSA/MAMBS^a^^,^^b^	1.8/0.7^a^	3.8/0.7^a^	2.9/0.9^a^
PLN^b^	n/d	n/d	0.31 ± 0.01
PLN–PEG^b^	n/d	n/d	0.33 ± 0.03

a From ref (30).

b Based on particle concentration.

c n/d means “not determined” as these were not tested.

Introducing AMBS into heparin, which removed the anticoagulation property (Figure 2b), further increased the growth inhibition potential of heparin, also against *P. knowlesi*, where unmodified heparin was much less effective than in the *P. falciparum* experiments (Figures 2d–f and S4). The higher activity of both unmodified and modified heparin against W2mef compared to 3D7 can be explained by their usage of more charged units for invasion (sialic acid-dependent invasion). Heparin was also previously confirmed to act at the merozoite invasion stage rather than influencing intraerythrocytic development of parasites.^14,38^ Hence, inhibitors using charge-based interactions are more efficacious against the W2mef strain as found previously.^30^ Obtaining more potent antimalarial activity of heparin while simultaneously removing the anticoagulation property is a key finding of this study and has the potential to bring heparin, when modified, back into the discussion for severe malaria treatment. Other heparin-related examples that have previously been tested extensively *in vitro* and *in vivo* include sevuparin, which also had lower anticoagulation potential than heparin and good invasion inhibition potential (EC_50_ at 5.2 μg/mL, 650 nM).^16^ Hence, our constructs PGA-AMBS and heparin-AMBS performed up to 5 to 10 times better than sevuparin on weight and molar bases, respectively. Considering relatively high plasma concentrations, with mean *C*_max_ between 56.7 and 149 μg/mL, that were achieved in the human trial with sevuparin,^16^ the decrease of the EC_50_ values by our modification could have significant implications. Modifying sevuparin with AMBS might be another interesting avenue to consider.

We also performed additional tests for PLNs (Figure 2f) and could demonstrate that PLNs without DSPE-PEG (EC_50_ at 0.73 ± 0.03 μg/mL of active AMBS polymer) had similar potency to our previously published PEGylated PLNs (EC_50_ at 0.80 ± 0.07 μg/mL of active AMBS polymer), otherwise made from the same components.^30^ Invasion inhibition activity of MAMSA/MAMBS micelles against various *P. falciparum* strains was also reported in that previous paper (EC_50_ values are reprinted in Table 1 for comparison). With respect to severe malaria, reducing iRBC numbers by acting at the key multiplication stage of reinvasion (the multiplication rate, corresponding to RBC reinfection is ca. 16 for *P. falciparum* in humans)^5^ could represent an interesting adjunctive therapeutic measure. Our nonanticoagulant, cytocompatible, and potent invasion inhibitory methoxybenzenesulfonated biopolymers and nanoparticles are promising candidates for such an application. Additional activity of our materials relevant to severe malaria was explored next.

### Antisequestration Activity of Methoxybenzenesulfonated Materials against CSA-Binder

**Figure 3:**
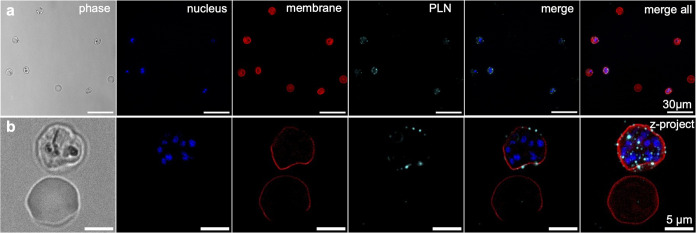
PLNs bind to the surface of CS2 parasite-infected RBCs (CSA-binder). (a) Widefield fluorescence overview image of nanoparticle (PLN-Atto647, cyan) interaction with CS2 iRBCs (nucleus in blue, WGA membrane stain in red). Scale bars, 30 μm. (b) Widefield fluorescence deconvolution imaging of the same sample as in panel (a). The first five images in panel (b) are middle slices of a deconvolved z-stack, while the image on the right is a max. intensity z-projection of the merged image. Scale bars, 5 μm. More images can be found in Figure S5.

Reducing iRBC sequestration is thought to be a second key strategy to help mitigate severe malaria conditions, including placental malaria, in the future. A potential application in the context of pregnancy-associated malaria requires disturbing the iRBC–CSA interaction. Hence, we first evaluated whether fluorescently labeled PLNs can interact with the surface of CS2 iRBCs, which is a parasite strain expressing VAR2CSA, the *Pf*EMP1 variant responsible for CSA binding. Indeed, when studying the interaction of PLN with a mixture of RBCs and CS2 iRBCs, we could clearly observe accumulation of PLNs on the iRBC membranes (Figures 3 and S5). The chemical nature of the hydrophilic block of PDLLA-AMBS contains many sulfonate groups and was designed to mimic heparin-like molecules.^30^ CSA is chemically related to heparin; hence, this is a likely explanation for the binding. Hep-AMBS and PGA-AMBS could not be labeled sufficiently through chemically conjugating a fluorophore to allow fluorescence imaging. This is likely due to quenching of the fluorophore by the high density of aromatic AMBS groups in close proximity.

**Figure 4:**
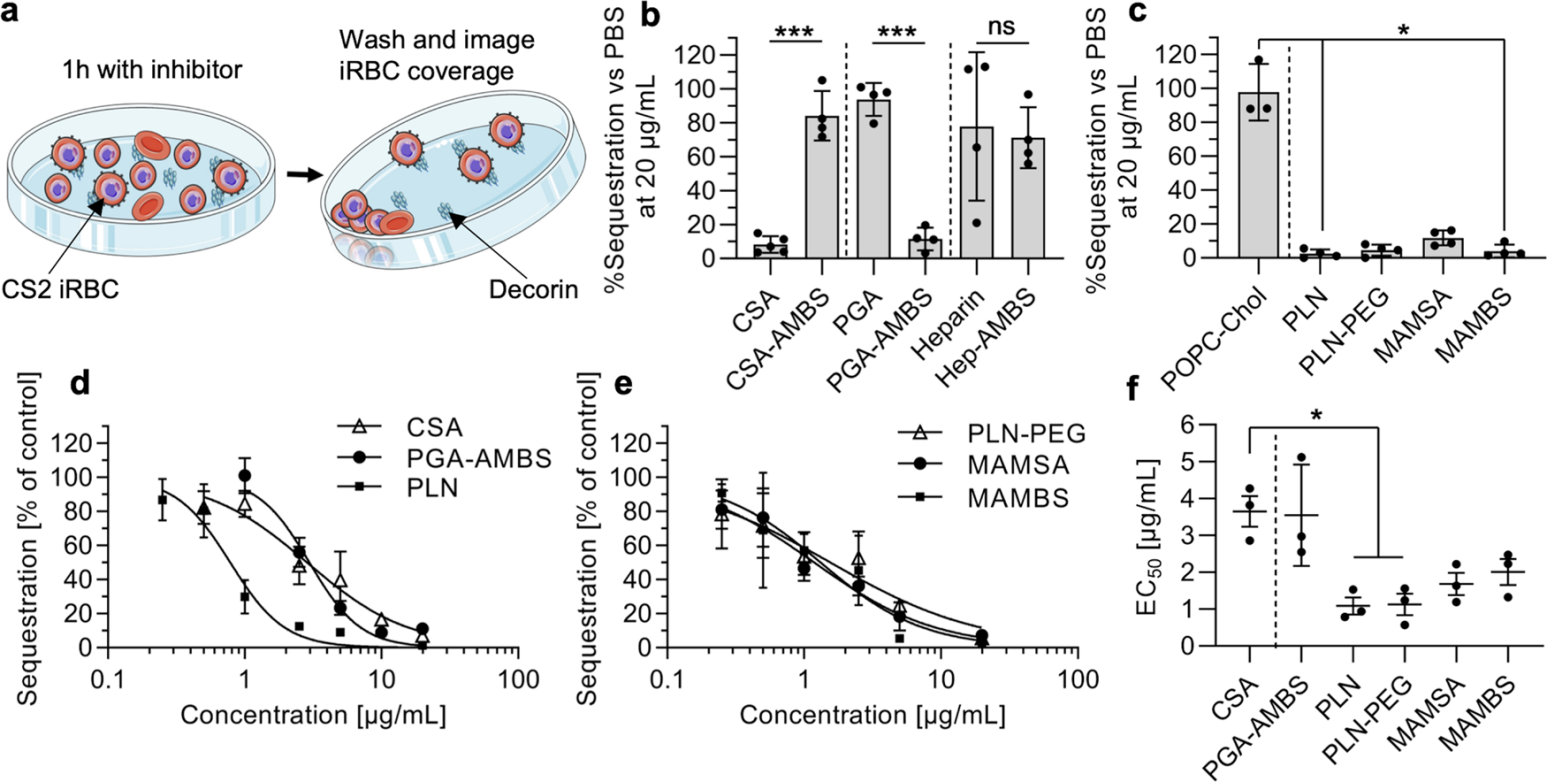
Antisequestration activity of methoxybenzenesulfonated soluble biopolymers and nanoparticles against CS2 parasite-infected RBCs (CSA-binder). (a) Schematic of the quantitative antisequestration assay. Schematic modified from Servier Medical Art Web site CC-BY. (b, c) Percentage sequestration after coincubation of CS2 iRBCs with soluble polymers (b) or nanoparticles (c) on decorin-coated plates *vs* PBS control when tested at 20 μg/mL (*N* ≥ 3 independent experiments with technical duplicates, one-way ANOVA with Šidák’s multiple comparisons test, ns = not significant, *P < 0.05, ***P < 0.001). (d, e) Examples of full dose–response curves for antisequestration experiments (as in panels (a–c)) (mean and range of *N* = 1 independent experiment with technical duplicates). (f) EC_50_ values obtained from dose–response curves (as shown in panels (d, e)) using *P. falciparum* strain CS2 (*N* = 3 independent experiments with technical duplicates, one-way ANOVA with Šidák’s multiple comparisons test, *P < 0.05). For nanoparticles, the amount of active polymer (AMSA or AMBS-modified) was used.

The observed interaction of PLNs with the CS2 iRBC surface was next hypothesized to function similarly to soluble CSA in inhibiting subsequent interaction with CSA receptors.^9^ To test this, we performed antisequestration experiments with decorin-coated 48-well plates (Figure 4). Decorin is a CSA-containing glycoprotein that has previously been used to coat surfaces for studying CS2 iRBC sequestration and inhibition.^39^ After incubation of knob-enriched CS2 iRBCs together with the above-developed AMBS-modified biomaterials and relevant controls for 1 h, the plates were washed, fixed, and stained, and the area of the wells coated with iRBCs was measured and compared to that of the PBS control.

When first evaluating the inhibitors at a fixed concentration of 20 μg/mL, we found interesting behaviors in our biomaterials (Figure 4b). Unmodified CSA was used as a gold-standard positive control and provided the expected antisequestration activity, in agreement with the literature.^9^ Interestingly, incorporating the AMBS modification in CSA, obtained *via* the same modification procedure as for PGA-AMBS and Hep-AMBS, reduced the antisequestration potential of CSA, suggesting that the specific interaction of CSA with CS2 iRBCs is dependent on the carboxylic acid residues in CSA. Conversely, AMBS-modified PGA revealed good antisequestration activity, while unmodified PGA was inactive, mirroring the growth inhibition data (Figure 2d,e). Heparin and heparin-AMBS both showed low activity, contrasting with the growth inhibition data in this case (Figure 2d–f). The inability of heparin to inhibit iRBC sequestration of a CSA-binder is in agreement with the literature.^40^ All of the nanoparticles, including PLNs with and without PEG and the polymer micelles from our previous study based on the AMSA-modified copolymer (MAMSA) and AMBS-modified copolymer (MAMBS),^30^ were all highly active in inhibiting CS2 iRBC binding to decorin receptors (Figure 4c). These interesting differences point toward a possibility to tailor inhibitors for CS2 iRBC antisequestration, as even small changes in the structures had a large effect on the antisequestration property.

The most potent inhibitors from above, specifically CSA, PGA-AMBS, MAMSA, MAMBS, PLN, and PLN–PEG, were then subjected to full dose–response evaluation with the same protocol using decorin-coated well plates and CS2 iRBC cultures (Figures 4d–f and S6). When comparing the EC_50_ values of our methoxybenzenesulfonated biomaterials to CSA, we found a significant improvement when using PLNs with and without PEGylation. Interestingly, for PLNs, the antisequestration activity (EC_50_ corresponding to 0.45 ± 0.10 nM particles) is of similar potency as growth inhibition (Figure 2f and Table 1). The improved activity in terms of antisequestration activity of nanoparticles *vs* soluble polymers, including gold-standard CSA or PGA-AMBS, could be due to multivalent interactions and creation of a larger gap between iRBCs and decorin. This study on CS2 iRBCs revealed the potential of methoxybenzenesulfonated materials, especially PLNs, to be evaluated further toward an adjunctive therapy for placental malaria. The best-performing structure (PLN) was taken forward to test against another parasite strain using a different *Pf*EMP1 variant, causing another severe malaria condition.

### Antisequestration Activity of Nanoparticles against ICAM-1-Binding iRBCs

Cerebral malaria represents a devastating, severe malaria form associated with high mortality. Parasites expressing *Pf*EMP1 that are capable of ICAM-1 and endothelial protein C receptor (EPCR) binding on the endothelium are the key candidates responsible for most cases of cerebral malaria.^20,41^ In addition, ICAM-1 is overexpressed in the inflamed endothelium, which is another hallmark of severe malaria. Inhibiting and reversing these *Pf*EMP1-endothelium interactions to liberate sequestered iRBCs from the brain vasculature could have a significant impact as an adjunctive treatment for patients with cerebral malaria. After the encouraging results of potent CS2 iRBC sequestration inhibition by PLNs (Figure 4), we continued to test their activity against another parasite line. ItG iRBCs express ITvar16, a *Pf*EMP1 variant with high affinity for ICAM-1.^24,42^

**Figure 5:**
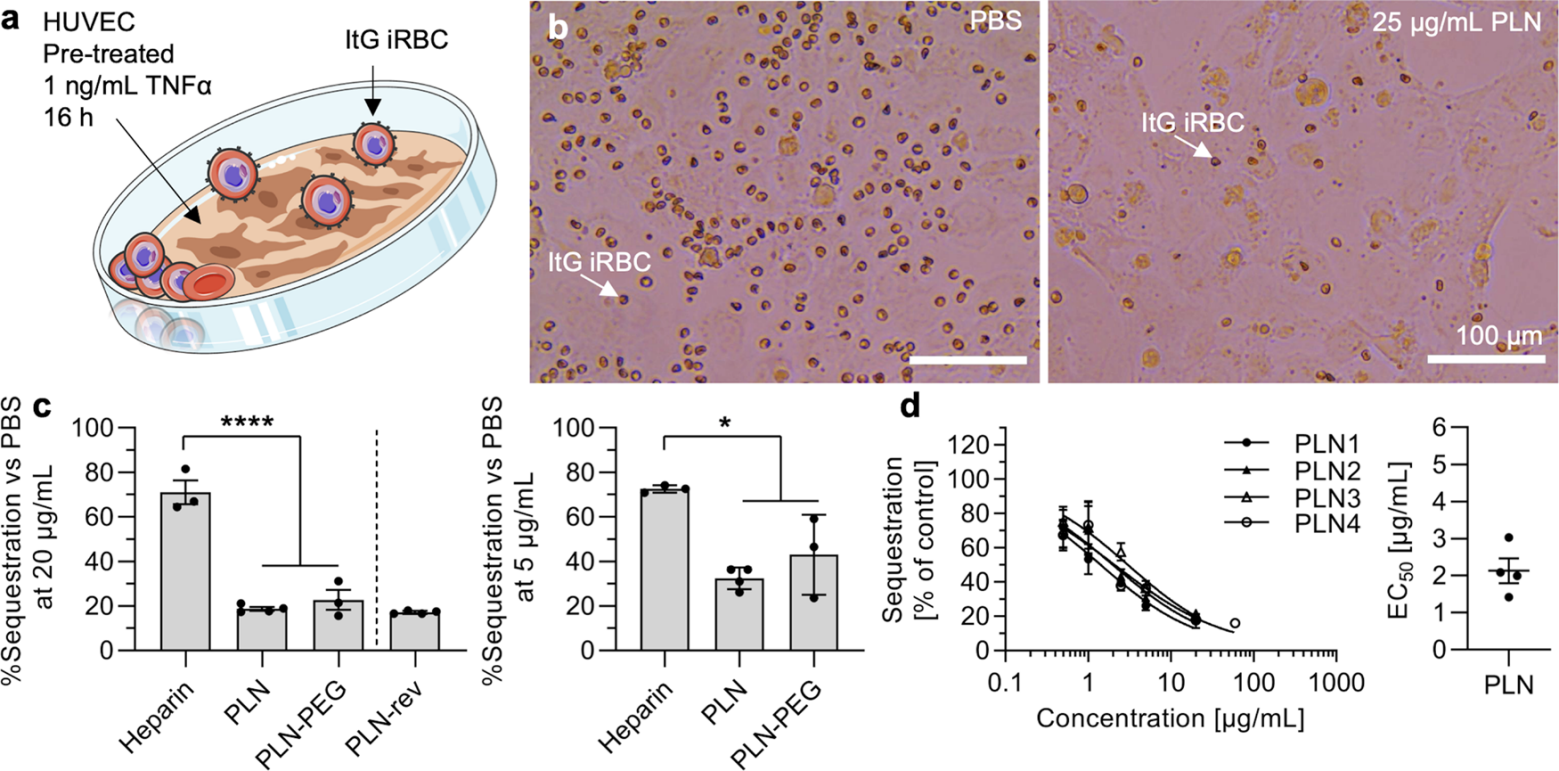
Antisequestration activity of PLNs tested against ItG-infected RBCs (ICAM-1-binder) incubated on HUVECs. (a) Schematic of the quantitative antisequestration assay using a HUVEC endothelial cell layer and ItG iRBCs. Schematic modified from Servier Medical Art Web site CC-BY. (b) Brightfield micrographs showing ItG iRBCs bound to HUVECs under inflammatory conditions (pretreated HUVEC with 1 ng/mL TNF-α for 16–18 h) after incubation for 1 h and washing with and without PLNs. Scale bars: 100 μm. Overview images and repeat on ICAM-1 receptor-coated well plates can be found in Figures S7 and S8. (c) Percentage sequestration after coincubation of ItG iRBCs with nanoparticles (rev refers to reversal of binding, adding the particle only during the washing steps) on HUVECs (pretreated with 1 ng/mL TNF-α for 16 h) *vs* PBS control when tested at 20 and 5 μg/mL, respectively (*N* ≥ 3 independent experiments with technical duplicates, one-way ANOVA with Šidák’s multiple comparisons test, *P < 0.05, ****P < 0.0001). (d) Examples of full dose–response curves for antisequestration activity of PLNs (same as in panel (c)) (mean and range of *N* = 1 independent experiment with technical duplicates) and corresponding EC_50_ values (*N* = 4 independent experiments with technical duplicates).

Therefore, we first performed an experiment analogous to the above CS2 iRBC-decorin setup but now using recombinant ICAM-1 immobilized on 48-well plates and switching to the parasite line ItG. The activity of PLNs and PEG–PLNs against ItG iRBC sequestration was confirmed in this setup (Figure S7). To better mimic the *in vivo* situation, including the inflammatory environment, we moved on to evaluate antisequestration activity on endothelial cells. We cultured HUVECs and added 1 ng/mL TNF-α 16–18 h prior to the experiment to simulate inflammation. This represents a common sequestration model, and these culture conditions were previously found to induce overexpression of ICAM-1 on HUVECs and promote ItG iRBC sequestration.^24,43−45^ When testing inhibition of ItG iRBCs binding to ICAM-1-expressing HUVECs, we again found potent activity of our PLNs, while the heparin control only demonstrated a modest effect (Figure 5).

The EC_50_ of 2.1 ± 0.3 μg/mL for the PLNs corresponding to 0.89 ± 0.14 nM particles (Figure 5d and Table 2) was in a range similar to that of the experiments with CS2 inhibition on decorin (Figure 4) and ICAM-1 receptor-coated dishes (Figure S7). Importantly, first allowing ItG iRBCs to adhere to HUVECs for 1 h and adding PLNs only during the washing steps (contact time of only about 5 min) efficiently reversed the interaction (Figure 5c). Liberating previously bound iRBCs from the endothelium is thought to be the most important property of an antisequestration adjunctive therapy.

**Table 2:** Comparison of Selected Anionic Inhibitory Components against *P. falciparum* iRBC Cytoadhesion (from the Literature and Figure 5)

inhibitor name	concentration [μg/mL]	concentration [nM]	inhibition [%]	cell type	parasite line	refs
CSA	∼3	60	50	CHO-K1	FCR3csa	(46)
CSA	100	2000	∼15	SBEC 1D	PA-ICAM-1	(47)
sevuparin	100	∼12500	∼75	HDMEC	clinical isolates	(48)
heparin	400	∼5600	∼60	HUVEC	ItG (ICAM-1)	(45)
PI-88	∼10	4000	∼40	CHO-K1	3D7csa	(49)
dextran sulfate	∼10	20	50	CHO-K1	FCR3csa	(46)
PLN	2	0.9	50	HUVEC	ItG (ICAM-1)	Figure 5

In comparison to our data, sevuparin required ≥100 μg/mL to inhibit sequestration on human dermal microvascular endothelial cells (HDMECs);^48^ hence, it was much less effective than our PLNs (Figure 5d and Table 2). Our PLNs also reveal various advantages over soluble CSA, as our nanoparticles were highly potent against sequestration of CSA- and ICAM-1-binding iRBCs, in addition to the merozoite invasion inhibition potential described above (Figure 2). In contrast, CSA efficiently inhibits CSA-mediated iRBC sequestration, but it is inactive against iRBC cytoadherence to ICAM-1 expressing cells (Table 2, FCR3csa *vs* PA-ICAM-1),^46,47^ and CSA does not inhibit merozoite invasion.^17^ The herein confirmed limited activity of heparin against ItG sequestration is also in agreement with the literature, as only 60% inhibition was achieved at a high concentration of 400 μg/mL heparin when tested with the same system as described here (ItG parasites on TNF-α treated HUVECs).^45^ In another study, heparin (ca. 500 μg/mL) did not reverse or inhibit ItG binding to ICAM-1 expressing cells.^50^ On the other hand, small molecules identified by a screen against ICAM-1- and CSA-binders, yielded ca. 80% inhibition of iRBC binding to receptor-coated dishes but only at high concentrations (EC_50_ at low to mid μM range) for both receptors.^22^ Despite a more recent small molecule screen identifying more potent inhibitors of ICAM-1 binding of iRBCs (EC_50_ at ca. 350 nM), reversal of the interaction, which is more clinically relevant, was not possible with these compounds.^23^

Hence, our finding that PLNs inhibited and reversed binding of an ICAM-1-binding parasite strain (ItG) at relatively low concentrations (EC_50_ at 2.1 ± 0.3 μg/mL, corresponding to 230 ± 33 nM active polymer, and 0.89 ± 0.14 nM particles), when tested in a relevant setting with endothelial cells, represents an important step toward creation of a universal sequestration inhibitor. Again, the higher activity of nanoparticles compared to soluble polymers for antisequestration might be attributed to multivalent interactions with iRBCs and the introduction of more spacing between iRBCs and endothelial cells. This is in agreement with a previous literature which shows that higher-molecular-weight soluble polymers show higher activity compared to their lower-molecular-weight counterparts (dextran sulfate 8 kDa *vs* 500 kDa) against sequestration.^47^ Therefore, the use of nanoscale constructs to mitigate severe malaria infection is a worthwhile avenue of research toward advancing strategies that reduce the high morbidity and mortality associated with this life-threatening condition.

## Conclusions

We have presented here the chemical modification of soluble biopolymers, including PGA and heparin, and nanoparticles with AMBS to improve the antimalarial and antisequestration activity of these materials. Reduction of parasite numbers and removal of sequestered infected cells from blood vessel walls are considered to be two important strategies in the development of an adjunctive therapy for severe malaria. The straightforward AMBS modification performed herein, only using a cheap commercial molecule and well-known EDC-coupling, turned all of the described structures more inhibitory in terms of RBC invasion inhibition, while ensuring negligible anticoagulation activity. When testing against another feature of severe malaria, *i.e.*, the binding of iRBCs to endothelial receptors, it was established that the AMBS-modified nanoparticles provided potent activity in inhibiting and reversing this sequestration process. Importantly, the nanoparticles were functional against two different parasite strains, a CSA-binder and an ICAM-1-binder, which are associated with placental malaria and cerebral malaria, respectively. Targeting different *Pf*EMP1 variants with the same construct is beneficial as it might allow broad-spectrum applicability against various strains using the same inhibitor. Future work is necessary to test activity under physiological flow to confirm suitability in a more realistic scenario.^51^ Our previous finding that incorporation of PEGylated lipids in this nanoparticle formulation extended blood circulation time,^30^ together with the antisequestration activity found herein also for this nanoparticle version (PLN–PEG), provides supportive arguments for the development of a nanoscale inhibitor. Further, a nanoparticle-based strategy allows straightforward combination with controlled release of additional drugs to adjust the overall treatment response.^32^ Hence, the findings reported herein represent an important step toward optimizing biopolymers and nanoparticles toward a potentially transformative severe malaria adjunctive therapy.

## Materials and Methods

### Nanoparticle Synthesis

Our previously developed protocol was employed to modify the copolymer and formulate nanoparticles.^30^ In brief, 10 mg/mL of the copolymer PDLLA-*b*-PAA (Poly(d,l-lactide-*bloc*k-acrylic acid), 9 kDa–9 kDa, Sigma-Aldrich, 802190) in MES buffer (0.5 M MES (Sigma-Aldrich) and 0.25 M NaCl (VWR), pH 6.0) were vigorously stirred for 30 min and subsequently ultrasonicated (bath) for 45 min. Per 10 mg of copolymer, 21.4 mg of AMBS (5-amino-2-methoxybenzenesulfonic acid, 1.5 equiv with respect to AA repeating units, ChemCruz, SC-233225), or 18.4 mg aminomethanesulfonic acid (AMSA, 2.5 equiv, Sigma-Aldrich, 127442) was added and dissolved. Next, aliquots of 0.5 eq of EDC-HCl (*N*-(3-(dimethylamino)propyl)-*N*-ethylcarbodiimide hydrochloride, 6.6 mg, Sigma-Aldrich, E7750) were added every 0.5–1 h, a total of 8 times, while stirring vigorously at room temperature and sonicating the solution for 1 min before each addition. To recover the micelles (MAMSA and MAMBS), 1 mL of modified sample was passed through a PD MidiTrap column (GE Healthcare) equilibrated in phosphate buffer (0.1 M phosphate (Sigma-Aldrich), 0.05 M NaCl (VWR), pH 7.4) and then a 30 cm Sepharose 6B column (Sigma-Aldrich, 6B100) equilibrated in phosphate-buffered saline (PBS, Sigma-Aldrich, D8537). To recover the copolymer for subsequent PLN assembly, 5 mL of modified sample was purified and desalted with 5 PD MidiTrap columns (GE Healthcare) equilibrated in phosphate buffer (0.1 M phosphate (Sigma-Aldrich), 0.05 M NaCl (VWR), pH 7.4), and 3 PD 10s (GE Healthcare) equilibrated in water. After concentrating the sample to about 3 mL using Amicon ultracentrifuge devices (100 kDa, Sigma-Aldrich), it was run through a cation-exchange column (AG-50W-X8, H+ form, Bio-Rad, 1435451, ca. 3–4 mL packed bead volume, washed with water). The obtained aqueous sample (PDLLA-AMBS) was sterile-filtered (0.22 μm syringe filter) and freeze-dried, giving a white fluffy powder.

PLNs (PLN-Mid) were assembled by mixing 5.4 mg of 1-palmitoyl-2-oleoyl-glycero-3-phosphocholine (POPC, Avanti, 850457P-200 mg), 2.7 mg of cholesterol (Sigma-Aldrich, C8667–5G), and 11.4 mg of PDLLA-AMBS. For PLN-Low and PLN-High, the amount of both the vesicle-forming POPC and cholesterol was doubled or halved, respectively, while keeping 11.4 mg of PDLLA-AMBS constant. To formulate PEGylated nanoparticles (PLN–PEG), 17.2 mg of 1,2-distearoyl-*sn*-glycero-3-phosphoethanolamine-*N*-[methoxy(poly(ethylene glycol))-5000] (DSPE-PEG5k, Laysan Bio, MPEG-DSPE-5000–1g) was included in the above PLN-Mid mixture. To fluorescently label the PLNs, DSPE-Atto647 (0.1 mg, AttoTech, AD647N-161) was included in the PLN-Mid mixture. The components were dissolved in 100 μL of ethanol each, combined, and briefly heated to obtain a clear solution. This mixture was subsequently injected rapidly into 0.6 mL of vigorously stirred phosphate buffer (0.1 M phosphate, 0.05 M NaCl) and pH adjusted to ca. 7.2 to 7.4 with drops of 2 M NaOH. A N_2_ stream was then used to evaporate the ethanol, and the sample was next run through a 30 cm column loaded with Sepharose 2B-CL (Sigma-Aldrich, CL2B300) equilibrated in PBS. The samples were subsequently sterile-filtered (0.22 μm syringe filters) and concentrated with Amicon ultracentrifuge devices (100 kDa, Sigma-Aldrich), if required. The concentration (μg/mL) of the sulfonated polymer in the final samples and the conversion to nM particle concentrations was obtained through a combination of Farndale microassays and fluorescence correlation spectroscopy (FCS) as described in detail in our previous paper.^30^

### Biopolymer Modification

The above method for PDLLA-*b*-PAA modification was also employed for polyglutamic acid (PGA, Sigma-Aldrich, P4761, 20.5 kDa), heparin (Sigma-Aldrich, H3393, 18 kDa), and chondroitin sulfate A (CSA, Sigma-Aldrich, C9819) modification. 10 mg/mL PGA, 20 mg/mL heparin, or 20 mg/mL CSA was prepared in MES buffer. 21.4 mg of AMBS or 19.4 mg of AMSA was added and dissolved per mL of sample. Eight aliquots of 6.6 mg of EDC-HCl were added over a period of 6–8 h, while sonicating the solution for 1 min before addition of a new aliquot. The samples were purified by sequential SEC using first PD MidiTrap column (GE Healthcare) equilibrated in phosphate buffer (0.1 M phosphate (Sigma-Aldrich), 0.05 M NaCl (VWR), pH 7.4) and second PD 10 (GE Healthcare) equilibrated in PBS. The samples were passed through 0.22 μm syringe filters for sterilization. The degree of modification was determined using UV–vis spectroscopy (SpectraMax M5, Molecular Devices) using the characteristic AMBS peak and assuming no loss of PGA or heparin during purification. The degree of modification was confirmed by ^1^H NMR in D_2_O using a JEOL 400 MHz spectrometer, assuming 1 available COOH per heparin disaccharide unit.

### Dynamic Light Scattering (DLS) and ζ-Potential Measurements

DLS measurements (*n* = 3) were performed on a Malvern Zetasizer Nano-ZS. 70 μL of nanoparticle sample in PBS was typically used in single use microcuvettes. For ζ-potential measurements, 950 μL of 0.3 M sucrose was mixed with 50 μL of purified nanoparticle sample in PBS and run on the same machine (*n* = 3).

### Fluorescence Correlation Spectroscopy (FCS)

FCS was performed on a commercial LSM 880 (Carl Zeiss, Jena, Germany), and data analysis was conducted with PyCorrfit program 1.1.6.^52^ A dilution series of Alexa647 in PBS was used to calibrate the confocal volume. All measurements were performed at 37 °C, correcting the diffusion coefficient for the higher temperature: Alexa647 in PBS (*D* = 4.42 × 10^–6^ cm^2^/s at 37 °C, *D* = 3.3 × 10^–6^ cm^2^/s at 25 °C).^53^ A HeNe laser (633 nm excitation), a 40× C-Apochromat water immersion objective (NA 1.2), and appropriate filter sets were selected. Measurements were performed 200 μm above the ibidi eight-well glass plate (80827, ibidi, Germany) using 5 μL of sample droplets. For each sample, 25 × 5 s intensity traces were recorded and autocorrelated. The following one component fit (*G*_1comp_ (τ)) was employed, with τ_D_ being the diffusion time, τ_trip_ is the triplet time (fixed between 1 and 10 μs) of triplet fraction *T*, *N* is the effective number of diffusing particles in the confocal volume, and SP is the structural parameter (fixed to 5).
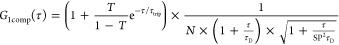
Hydrodynamic diameters (*D*_h_) were calculated from diffusion coefficients (*D*) and employed the Einstein–Stokes equation.

### Single-Particle Automated Raman Trapping Analysis (SPARTA)

Nanoparticles were characterized with SPARTA, which uses the combination of Raman spectroscopy and optical trapping to measure single-particle chemical information at the population level.^33^ Measurements were performed on the SPARTA 2.0 setup, as previously described.^54^ Briefly, a 785 nm laser (200 mW, Cheetah, Sacher Laser Technik, Germany) was directed through a custom confocal microscope fitted with a 63x/1.2 NA water immersion lens (W Plan-Aprochromat, Zeiss, Oberkochen, Germany) to form the optical trap and simultaneously excite Raman scattering from the trapped particle. This Raman signal was directed onto a spectrograph (HoloSpec-F/1.8-NIR, Andor, U.K.) coupled with a thermoelectrically cooled (−60 °C) back-illuminated CCD camera (iDus 416ALDC- DD, Andor, U.K.). 200 μL amount of nanoparticle suspension was pipetted onto a thickness #0 glass coverslip affixed to a standard microscope slide, and the droplet was interfaced with the objective. The sample was measured with an exposure time of 10 s per particle and a laser disabling time of 1 s between particles. DPBS was measured with the same measurement parameters for background subtraction. The Raman spectra were preprocessed using custom Matlab scripts for cosmic spike removal, spectral response correction (785 nm reference standard National Institute of Standards and Technology), background subtraction, baseline correction, smoothing, and normalization.

### Anticoagulation Assays

Antifactor Xa tests (Iduron, Anti-Xa Heparin XAE-200) were performed following manufacturer’s instructions but scaling down all of the volumes. 25 μg of Factor Xa (EXA-25) and 5 IU antithrombin (PAT-5) were mixed with 10 mL of Tris buffer (0.05 M Trizma Base, 0.175 M NaCl, 0.1% (w/v) PEG6000, 0.0075 M EDTA, pH 8.4), while 5 mg of Xa substrate (SXE-5.0) was dissolved in 10 mL of ddH_2_O. Commercial heparin (Sigma H3393, 189 USP/mg) was prepared in PBS and serially diluted to act as calibration series. Protein low-bind 1.5 mL Eppendorf tubes were placed in a Thermomixer set to static at 37 °C. Calibration and experimental samples (10 μL) were first combined with 40 μL of Tris buffer and equilibrated at 37 °C for 2 min. This was followed by sequential addition of 50 μL of antithrombin, 50 μL of Factor Xa, 50 μL of Xa substrate, and in the end 50 μL of 20% (v/v) acetic acid while incubating the tubes for 2 min at 37 °C after each addition. The negative control consisted of PBS only, while for the blank, all of the reagents were combined the wrong way around, starting with the acetic acid solution. Spike controls of samples mixed with known heparin amounts were included to test for potential nonspecific assay inhibition by the samples. Finally, a total volume of 300 μL per sample was transferred into a transparent flat bottom 96-well plate. A plate reader (SpectraMax M5, Molecular Devices) was used to measure absorbance from 350 to 500 nm, while the absorbance at 405 nm was employed for the calculations. Due to development of some turbidity in some samples, an exponential decay function was fitted to the scattering contribution within the curves and subtracted from the data to correct the baseline. Each sample was tested in duplicate per experimental repeat.

### RAW Cell Viability Tests

Cytocompatibility was studied according to the protocol in BS ISO 19007:2018.^55^ DMEM medium (high glucose) containing fetal bovine serum (FBS, Gibco, 10% (v/v)) and P/S (1% (v/v), Sigma-Aldrich) was prepared to culture RAW 264.7 cells. For the experiment, 15,000 RAW 264.7 cells/well were seeded in 96-well plates according to the suggested plate setup BS ISO 19007:2018. After incubating the plates at 37 °C for 24 h, the spent medium was replaced with 180 μL of new medium and 20 μL of sample solution in PBS or PBS only (100% viability control). After another incubation for 24 h at 37 °C, the supernatants were again removed and replaced with 120 μL of a mixture of MTS (317 μg/mL, Abcam, ab223881) and PMS (7.3 μg/mL, Sigma-Aldrich, P9625) in phenol-red-free RPMI medium. Absorbance at 490 nm was measured using a plate reader (SpectraMax M5, Molecular Devices) after incubation of the plates for 1–2 h at 37 °C in the dark.

### Malaria Parasite Culture and Growth Inhibition Assays

*P. falciparum* strains 3D7, D10, W2mef, and CS2 were cultured in human O^+^ RBCs as described elsewhere^56^ using RPMI-HEPES (Sigma-Aldrich, R5886) medium supplemented with 5 g/L Albumax II (Gibco),^57^ 0.292 g/L l-glutamine, 0.05 g/L hypoxanthine, and 0.025 g/L gentamicin (called malaria culture medium, MCM, while incomplete medium, ICM, refers to MCM without Albumax II). Synchronization was performed with 5% (w/v) sorbitol.^58^ The ItG parasite line was cultured in the same medium but replacing Albumax with 10% (v/v) human serum. *P. knowlesi* strain A1-H.1 was cultured in human O^+^ RBCs as described elsewhere and using RPMI-HEPES medium supplemented with 2 g/L dextrose, 0.292 g/L l-glutamine, 2.3 g/L sodium bicarbonate, 0.025 g/L gentamicin, 0.05 g/L hypoxanthine, 5 g/L Albumax II (Gibco), and 10% (v/v) equine serum (Life Technologies).^59^ Parasite stock cultures were kept static at 37 °C with a gas mixture of 90% N_2_, 5% O_2_, and 5% CO_2_.

Shaking culture growth inhibition assays were conducted as described elsewhere.^28^ In brief, 135 μL of parasite mix at 5% hematocrit and 1–2% parasitemia (synchronized trophozoite/schizont stage *P. falciparum*) in the above-described culture media were combined with 15 μL of test sample or PBS (100% growth control) in flat bottom 48-well plates. Stacked plates were surrounded with wet tissue paper in a gastight plastic box. After gassing the box with the mixture described above, the box was incubated at a tilt angle of about 15° under shaking at 185 rpm inside a cell culture incubator. The next day (typically 18–24 h later), 10 μL of each well was pipetted into U-bottom 96-well plate containing 200 μL of PBS. The plates were centrifuged, the supernatants were discarded, staining solution was added (200 μL 1/5000 dilution of SYBR Green (Invitrogen, S7563) in PBS), and incubated for 20 min at room temperature. Then, the plates were washed 3 times with 200 μL of PBS and parasitemia determined on a flow cytometer (plate reader, BD LSRFortessa II). EC_50-_curves were analyzed using QtiPlot.

### Malaria Antisequestration Trials on Receptor-Coated Dishes

For experiments with receptor-coated dishes, 48-well plates were coated with either 100 μL of 2 μg/mL decorin (CSA-containing glycoprotein, Sigma-Aldrich, D8428) or 10 μg/mL ICAM-1 (Sino Biological, 10346-H08H) in PBS for at least overnight in a humidified chamber in the fridge.^39,60^ The plates were washed twice with PBS and blocked with MCM for 1 h at room temperature and washed 3 times with PBS. CS2 and ItG parasite cultures were gelatin-floated to purify late-stage parasites with knobs on the surface from early stage parasites, knobless parasites, and uninfected RBCs according to a literature procedure.^56^ Purified late-stage parasites were washed 3 times with MCM (carbonate-free, pH 6.8 for CS2)^56^ or ICM (ItG). Mixtures of CS2 in MCM (carbonate-free, pH 6.8) at 6 Mio. iRBCs/mL and ItG in an ICM at 8 Mio. iRBCs/mL were prepared.^24,39,61^ 135 μL of these mixtures were added to each well of the 48-well plate and followed by 15 μL of PBS (100% sequestration control) or 15 μL of test sample in PBS. The plates were incubated under static conditions at 37 °C for 1 h. Then, the supernatants were pipetted away by tilting the plates to one side and using multichannel pipettes. 200 μL of MCM (carbonate-free, pH 6.8)/ICM was subsequently added from the opposite side to where the supernatants were pipetted off. The plates were not shaken but tilted to the other side again to pipette away the supernatants from the same side as before. This step was repeated at least 5 times (the last two were always with ICM (carbonate-free, pH 6.8)/ICM for CS2 or ItG, respectively). The plates were then emptied, air-dried, MeOH-fixed, and Giemsa-stained for further analysis.

### Malaria Antisequestration Trials on HUVECs

HUVECs were cultured in EGM-2 BulletKit medium (CC-3162, Lonza) without heparin. Cells were seeded into six-well plates by adding 1 mL of 2 × 10^6^ cells/mL to each well. The plates were incubated overnight and washed with culture medium to remove floating cells. HUVECs were cultured for one more day to form the endothelial layer. To create an inflammatory environment, 1 ng/mL TNF-α was added and incubated for 16–18 h prior to the sequestration experiment to overexpress ICAM-1 as done previously in ItG sequestration trials.^24^ The HUVECs were next washed with ICM. ItG parasites were gelatin-floated to collect iRBCs with knobs.^56^ Next, a cell mixture of 6–8 Mio. ItG iRBCs/mL (at 50–75% parasitemia) was prepared in ICM as previously done when testing other inhibitors.^24,61^ 0.95 mL portion of this mix was added per well together with 50 μL of PBS or 50 μL of test samples at various concentrations in PBS. The plates were incubated under static conditions at 37 °C for 1 h. Then, the plates were rotated horizontally by hand to lift off any unbound cells, the 1 mL mixture was taken up into a pipette from the right, holding the plate on a tilt angle, and readded into each well from the left, the plate swirled around again, and this was repeated 5 times in total. Then, the mixture was removed, and the wells were washed 5 times with ICM only, using the procedure of add, swirl, take away as described above. To test for reversal of iRBC binding, the nanoparticle solution was only added for all of the washing steps after the 1 h incubation time. The wells were then fixed with 4% (v/v) PFA and 0.4% (v/v) GA in PBS for 20 min and PBS-washed for further analysis.

### Microscopy and Analysis

Fluorescence images (including z-stacks) were recorded on a Nikon Ti Microscope with a 100x oil immersion objective. EpiDEMIC plugin in Icy (50 iterations) was used to deconvolve the z-stacks.^62^ Images were subsequently processed in Fiji. Brightfield imaging of Giemsa-stained iRBCs within 48-well plates and fixed iRBCs on HUVECs in 6-well plates was conducted on an EVOS microscope using a 10×/20× objective. Images were always taken just left of the center of the wells. Fiji was used to turn the images into a binary mask to measure the area covered by iRBCs, and all of the data was compared to the PBS control (set to 100% sequestration). For 6-well plate experiments, 3 random points just left to the center of the well were imaged per well and averaged. All of the data was again compared to the PBS control (set to 100% sequestration).

### Statistical Analysis

All of the figure captions explain the sample sizes, employed statistical tests, and post hoc analysis. Each caption also clarifies data normalization and representation. Growth inhibition and antisequestration EC_50_-curves were analyzed using QtiPlot (https://www.qtiplot.com). All other data was analyzed and plotted using GraphPad Prism 9.0.0.

## Supporting Information Available

Material characterization (UV–vis spectroscopy, ^1^H NMR); stability analysis (DLS, ζ-potential, UV–vis spectroscopy); anti-Xa assays (UV–vis spectroscopy); and parasite inhibition data and images (Figures S1–S8) (PDF)
