# Supplementary material for: Enhanced Antimalarial and Antisequestration Activity of Methoxybenzenesulfonate-Modified Biopolymers and Nanoparticles for Tackling Severe Malaria
Source: ACS Infect Dis. 2024 Jan 25;10(2):732–45. doi: 10.1021/acsinfecdis.3c00564 (PMC10862538; doi:10.1021/acsinfecdis.3c00564)
Supplement: Supplementary file 1 — id3c00564_si_001.pdf [file id3c00564_si_001.pdf]

SUPPORTING INFORMATION

Enhanced Antimalarial and Antisequestration Activity of Methoxybenzenesulfonate-Modified Biopolymers and Nanoparticles for Tackling Severe Malaria

Adrian Najer[a,b,], Junyoung Kim [a], Catherine Saunders[a], Junyi Che[a], Jake Baum[b,*], and Molly M. Stevens[a,c*]*

[a]Dr. A. Najer, Dr. J. Kim, C. Saunders, Dr. J. Che, Prof. M. M. Stevens
Department of Materials, Department of Bioengineering, and Institute of Biomedical Engineering, Imperial College London, London SW7 2AZ, UK.

[b]Dr. A. Najer, Prof. Jake Baum
Department of Life Sciences, Imperial College London, London, SW7 2AZ, UK.

[c]Prof. M. M. Stevens
Department of Physiology, Anatomy and Genetics, Department of Engineering Science, and Kavli Institute for Nanoscience Discovery, University of Oxford, OX1 3QU, Oxford, UK.

Corresponding authors E-mail: adrian.najer@kcl.ac.uk; jake.baum@unsw.edu.au; molly.stevens@dpag.ox.ac.uk

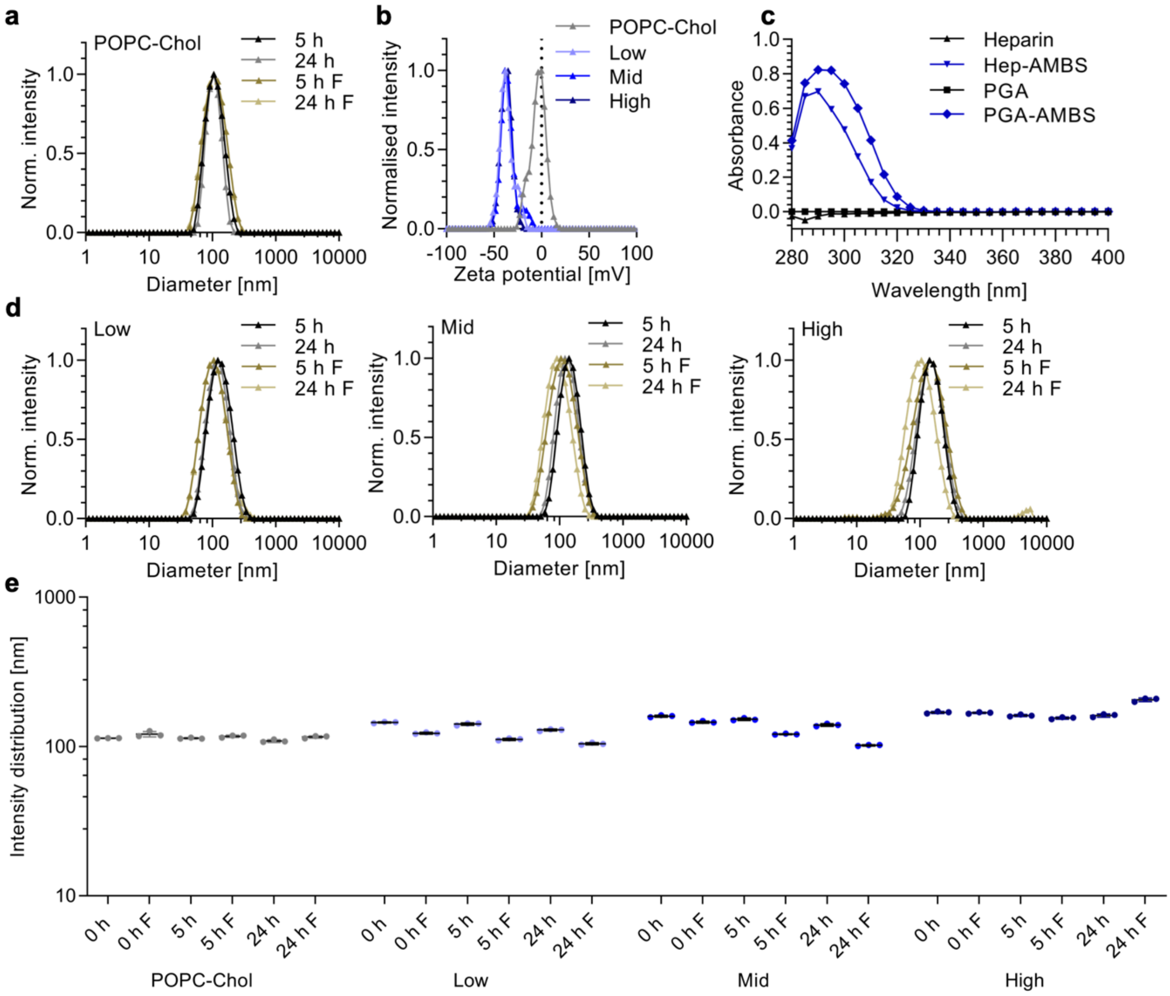

Figure S1. (a) Average DLS size distribution (intensity) of POPC-Chol control vesicles incubated over time in PBS (black/gray) +/- 10 v/v% FBS (F, green) (mean of technical triplicates). (b) Zeta potential distributions of POPC-Chol and PLNs (mean of technical triplicates), mean values from these curves appear in main Figure 1. (c) UV-Vis absorbance of solutions of heparin, heparin-AMBS, PGA, PGA-AMBS in PBS ($n = 1$). (d) Average DLS size distribution (intensity) of PLNs (Low/Mid/High) incubated over time in PBS (black/gray) +/- 10 v/v% FBS (F, green) (mean of technical triplicates). (e) Mean diameters from a,d (mean ± s.e.m., technical triplicates).

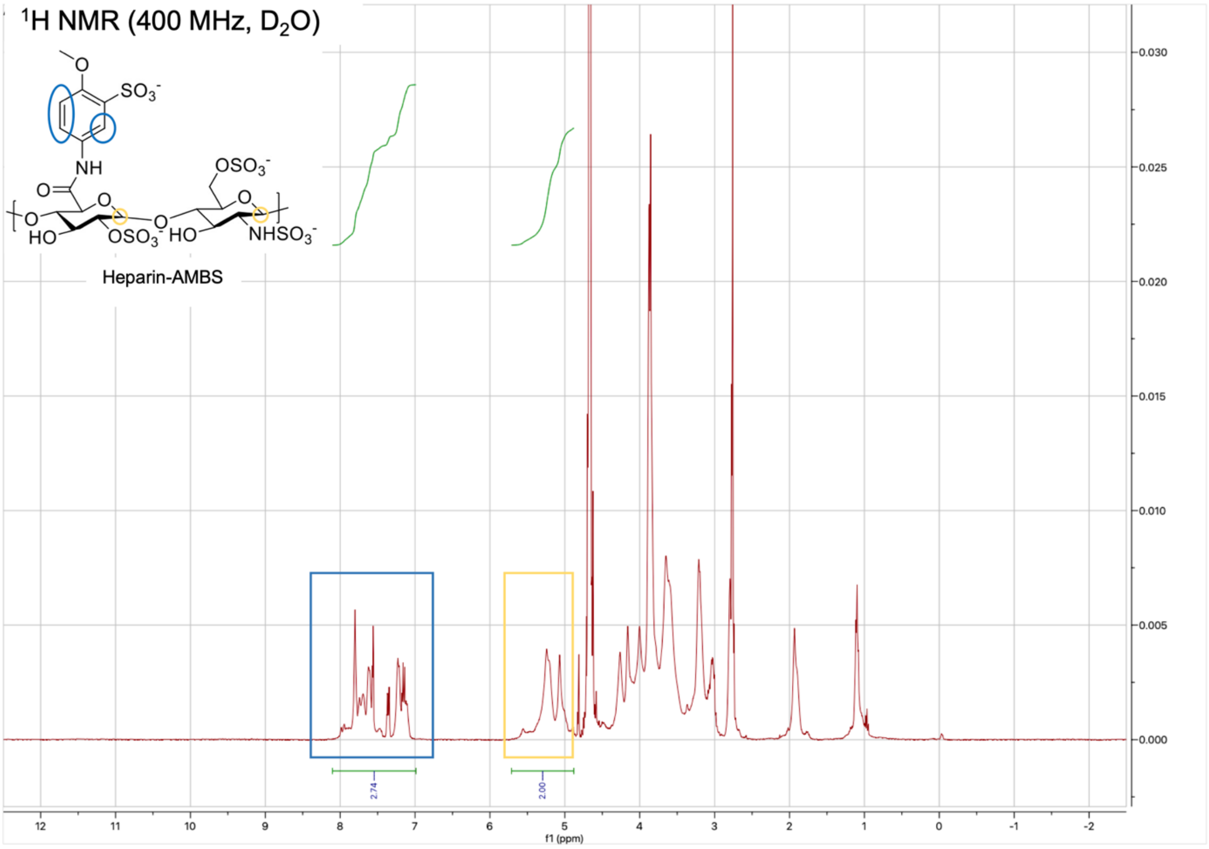

Figure S2. ^{1}H NMR of heparin-AMBS.

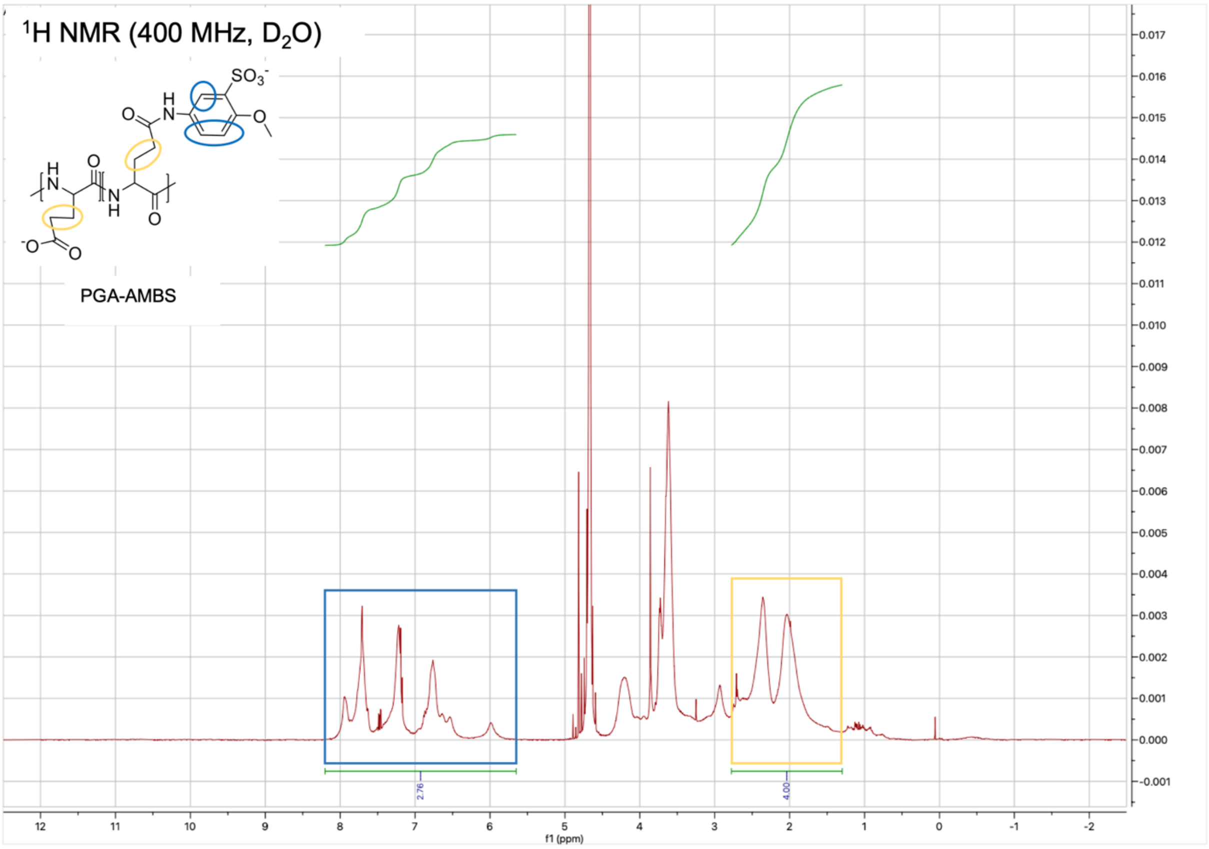

Figure S3. ^{1}H NMR of PGA-AMBS.

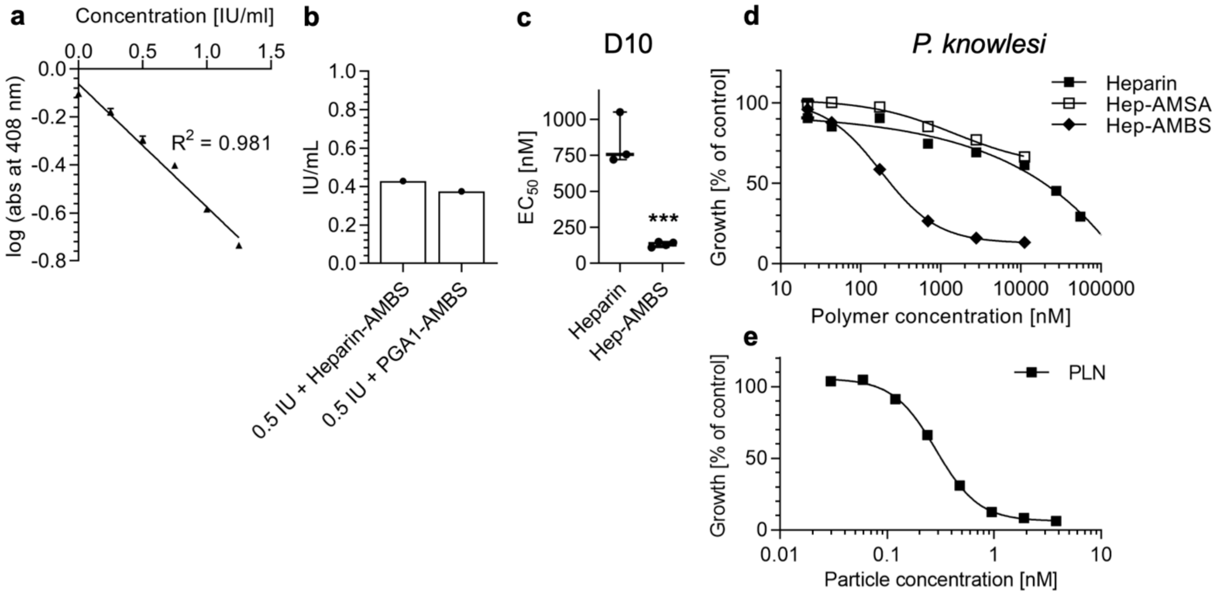

Figure S4. (a) Calibration curve for anti-factor Xa activity assay using an unmodified heparin dilution series (N = 1 independent experiment in duplicate). (b) Recovered anti-factor Xa activity of PGA-AMBS and Hep-AMBS when spiking with known 0.5 IU/mL unmodified heparin amount (N = 1 independent experiment in duplicate). (c) EC_{50} values obtained from dose-response curves for growth inhibition data using *P. falciparum* strain D10. ($N \geq 3$ independent experiments with technical duplicates, unpaired t-test, ***$P < 0.001$). (d) Dose-response curves for *P. knowlesi* A1-H.1 strain inhibition in suspension culture using heparin, heparin-AMBS, and heparin-AMSA (mean and range of N = 1 independent experiment with technical duplicates). (e) Example dose-response curve for *P. falciparum* 3D7 inhibition with PLNs tested in suspension culture assay (mean and range of N = 1 independent experiment with technical duplicates).

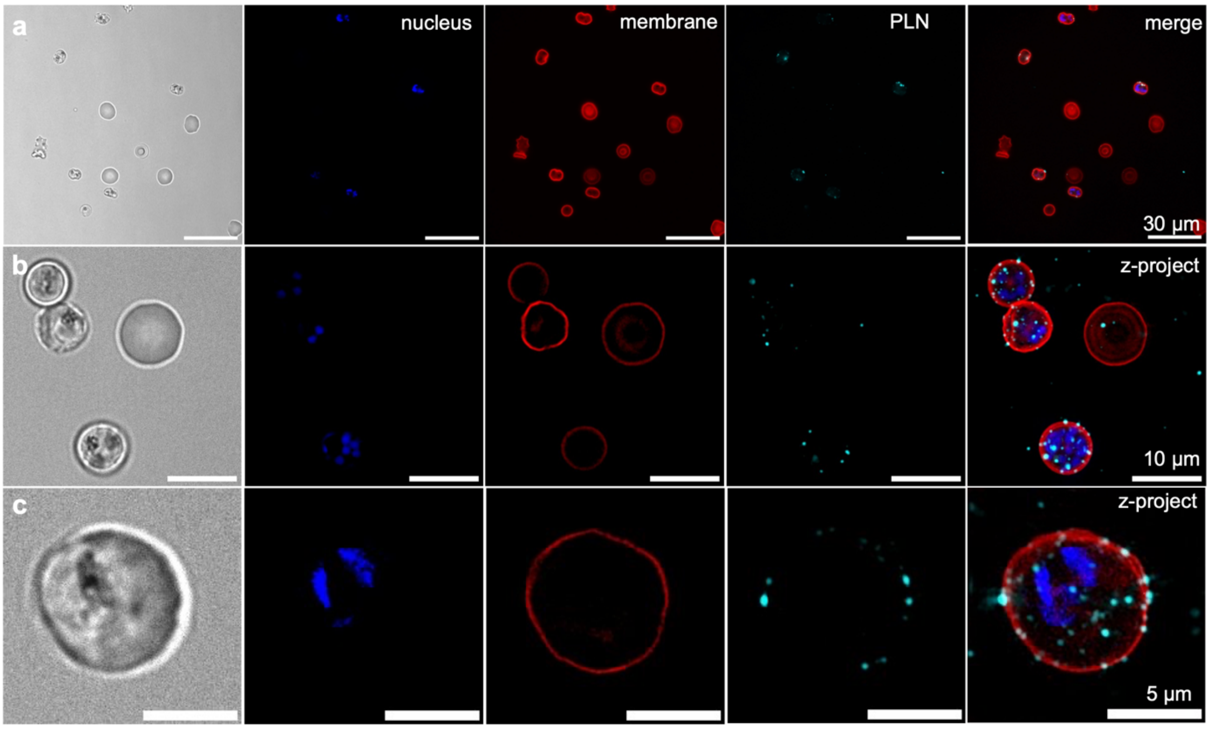

Figure S5. (a) Additional widefield fluorescence overview image of nanoparticle (PLN-Atto647) interaction with CS2 iRBCs (nucleus in blue, WGA membrane stain in red, PLNs in cyan). Scale bars, 30 µm. (b,c) Widefield fluorescence deconvolution imaging (middle slice of z-stack and max. intensity z-projection of merge) of same sample as in (a). Scale bars, 10 µm and 5 µm, respectively.

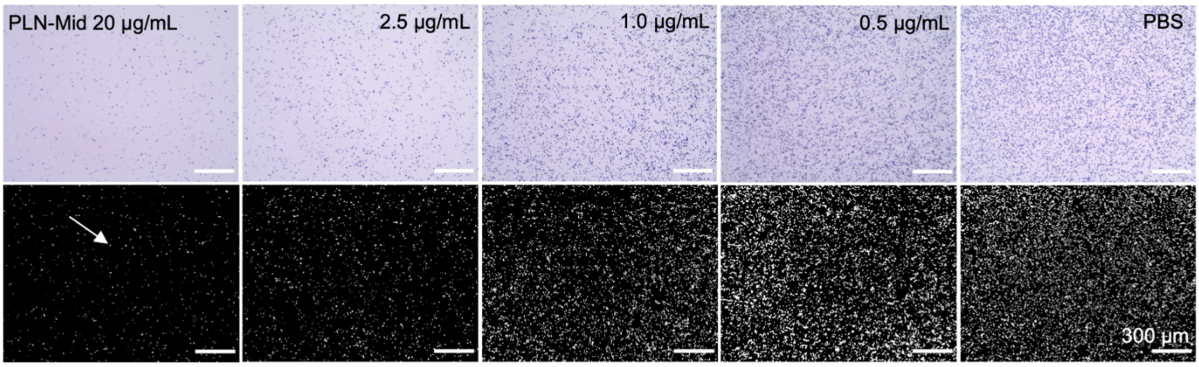

Figure S6. Brightfield images (top) and corresponding binary mask (bottom) used for relative sequestration quantification compared to PBS control. CS2 iRBCs binding to decorin-coated 48-well plates. Scale bars, 300 µm. The white arrow indicates a single iRBC.

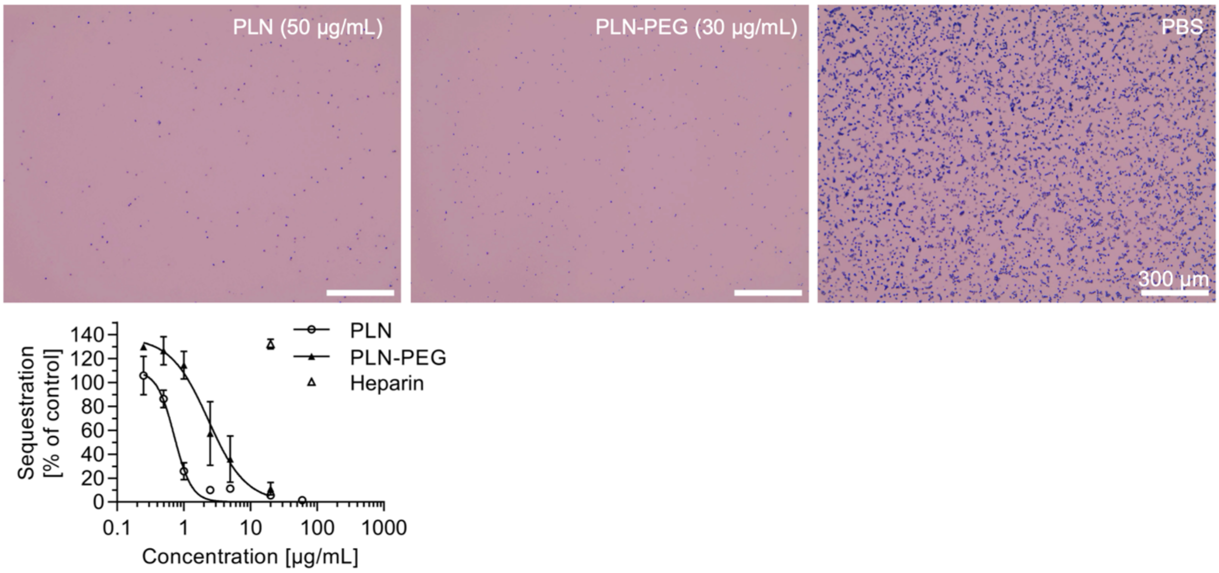

Figure S7. Brightfield images of ItG iRBCs binding to ICAM-1-coated 48-well plates. Scale bars, 300 µm. Full dose-response curves for anti-sequestration experiments with ItG iRBCs on ICAM-1 coated 48-well plates (same as in a) (mean and range of $N = 1$ independent experiment with technical duplicates).

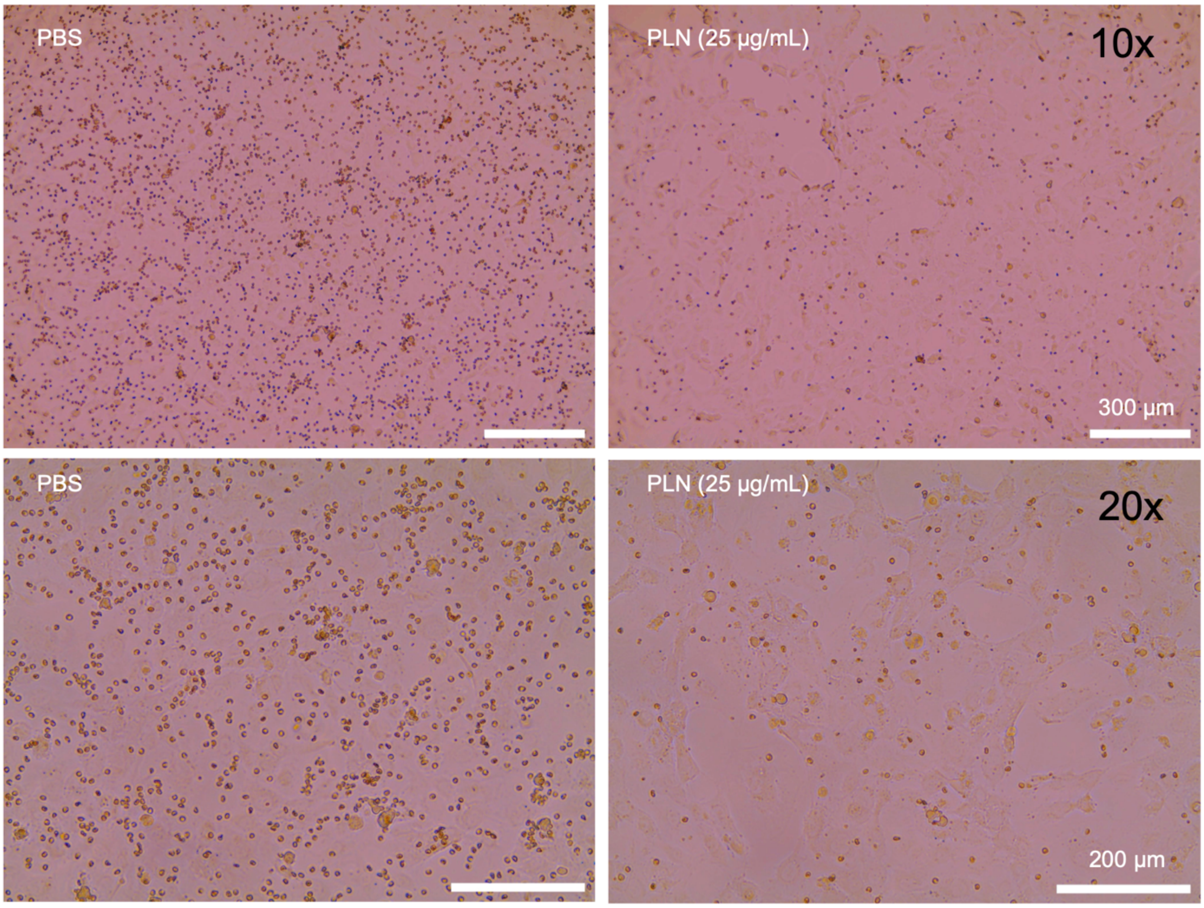

Figure S8. Brightfield images (10x and 20x magnification) of ItG iRBCs binding to TNF-α-treated (16 h) HUVEC monolayers in 6-well plates. Scale bars, 200 and 300 µm, respectively. Zooms of 20x images are shown in main Figure 5b.
